# Benign cystic mesothelioma of the peritoneum: a case report and literature review

**DOI:** 10.1186/1749-7922-8-43

**Published:** 2013-10-13

**Authors:** Hicham Elbouhaddouti, Abdesslam Bouassria, Ouadii Mouaqit, El Bachir Benjelloun, Abdelmalek Ousadden, Khalid Mazaz, Khalid Ait Taleb

**Affiliations:** 1 Department of Surgery, School of Medicine and Pharmacy of Fez, Sidi Mohammed Ben Abdallah University, University hospital HASSAN II, BP: 1893, Km2.200, Route de Sidi Hrazem FEZ 30000, Morocco

## Abstract

Benign cystic mesothelioma of the peritoneum (BCM) is an uncommon lesion with some 130 cases reported since the first case described by Smith and Mennenmeyer in 1979. It is a rare intra abdominal tumor occurring predominantly in women of reproductive age. Due to the rarity of this tumor, similarity of patient presentation, and comparable features on imaging, the diagnosis of this pathology is difficult, and is based on histological findings. This tumor is known for local recurrence. It's agreed that surgery is the only effective treatment, but there are no evidence-based treatment strategies for BCM.

## Introduction

Benign cystic mesothelioma of the peritoneum (BCM) is a rare intra abdominal tumor with a strong predilection for the peritoneum of pelvic organs. Symptoms are not specific, and the differential diagnosis is vast, including cystic lymphangioma, mucinous cystadenoma, cystic teratoma and pseudomyxoma retroperitonei. There are no evidence-based treatment strategies for BCM, and even if it is considered as a benign tumor, this tumor has a high local recurrence rate.

We report a new case of BCM, which appeared as a surgical emergency.

### Case report

**Figure 1:**
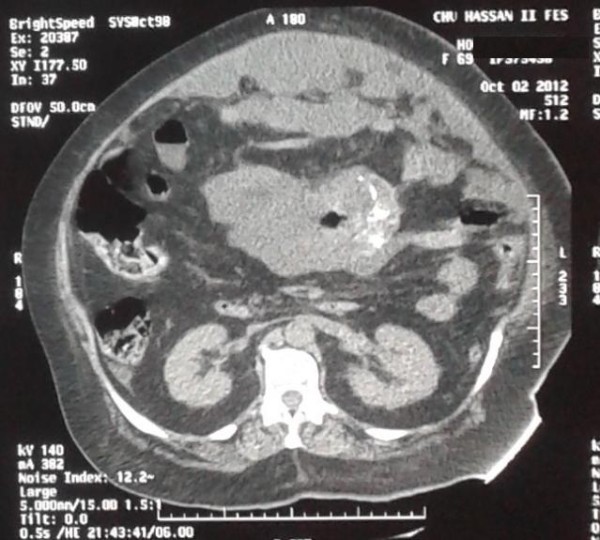
Large spherical multi-loculated cystic mass.

**Figure 2:**
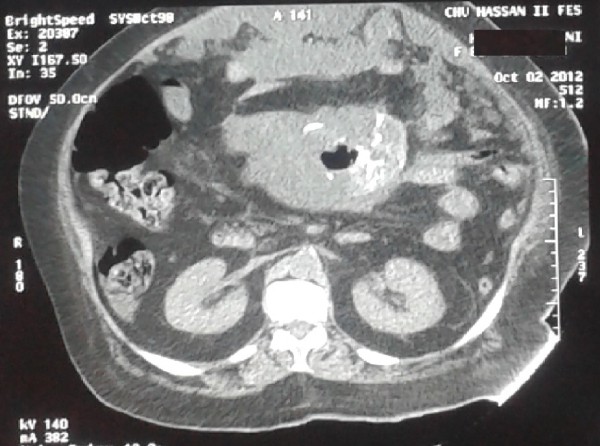
Suspicion of centro tumoral necrosis on CT scan.

A 71 year-old woman presented to the emergency department complaining of history of abdominal pain since 2 days accompanied by diarrhea. Four months prior to presentation, she noticed an increase in abdominal girth. Moreover, she developed occasional abdominal discomfort, which slowly increased frequency. The patient also developed symptoms of constipation and severe reflux which were not improved by taking laxatives and a proton pump inhibitor. Our patient was hemodynamically stable with temperature at 37.9°C, and blood pressure was 130/80 mmHg. Abdominal examination was marked by diffuse abdominal distension, and tenderness. Computed tomography (CT) scanning showed a large spherical multi-loculated cystic mass in the abdomen which was not-communicating to any abdomen viscera, occupying nearly the entire abdominal cavity, displacing the hole bowel (Figure 1). Furthermore, on CT scan, there was a strong suspicion of central tumor necrosis (Figure 2). Therefore, our patient was taken to operating theatre. Laparotomy was done. Intraoperative examination showed a cystic mass extending from the pelvis inferiorly to the liver. There was a significant peritoneal thickening, and a peritoneal effusion, with many cystic lesions that makes dissection and resection very difficult. The mass and some of the free-floating cysts were carefully harvested and removed for histological examination. Benign cystic mesothelioma was revealed in the pathology report.

Our patient made an excellent recovery, and she was discharged home after 6 days. Our patient was seen in out patient clinic at 1 month and 3 months. She had no functional complaints and kept a slight abdominal distension.

This study was performed according to the declaration of Helsinki and approved by the Local Ethical Committee.

## Discussion

Benign cystic mesothelioma of the peritoneum (BCM) was described first by Mennenmeyer and Smith [1]. It’s a rare pathological entity with about 130 cases reported in the literature [2,3] (Table 1). Several authors consider this tumor as benign [1,4], and it’s prognosis is excellent [5]. There is only one reported death from BCM on the literature: Raafat and al. reported a case of a 14 years-old patient who had a subtotal resection of the abdominal mass, and died 12 years after refusing surgery for recurrence [6]. Indeed, BCM has a high local recurrence rate [7], and this recurrence rate is higher in women (40 – 50%) than in men (33%) [8]. The etiology remains unclear, but it is well known that many inciting factors may promote hyperplastic and neoplastic changes in mesothelial cells. The suggested provoking factors are foreign fibres and dusts, inflammatory mediators, and mechanical injuries [9]. Proliferation and inward migration of peripheral mesothelial cells, proliferation and metaplasia of underlying connective tissue cells, and surface attachment and differentiation of free-floating mononuclear cells all have been postulated as the mechanism of mesothelial cell proliferation in pathological conditions [9]. This peritoneal lesion is characterized by the formation of multiple multilocular thin-walled cysts, which may form large intraabdominal masses [1]. The BCM affects women in 80% of cases, with an average age of 34 years [3]. The clinical presentation is unspecific: it is usually abdominal pain, increased abdominal girth and constipation. Physical examination revealed abdominal distension, abdominal tenderness or a palpable mass [10]. Imaging modalities that can be used include abdominal ultrasound (US), CT scan, or magnetic resonance imaging (MRI). They allow to visualize the lesion, but not to differentiate it from other cystic lesions of the peritoneum [11], especially lymphangiomas [9]. Laparoscopy remains the best diagnostic tool because it enables to perform biopsies and to establish the definitive diagnosis [12]. There are no evidence-based treatment strategies for BCM, but surgery, with complete enucleation of the cyst to prevent recurrence and possible malignant transformation remains the mainstay of treatment. However, some researchers advocate aggressive surgery followed by heated intraperitoneal chemotherapy (HIPEC) [12]. Indeed, for a long time, the treatment consist of full excision of the lesions (debulking surgery) [7]. Currently, some teams recommend aggressive surgery (extended peritonectomy) followed by HIPEC [3,13]. Two series are available on the results of extended peritonectomy followed by HIPEC. In the first one [13], 5 patients were asymptomatic, and 4 showed no recurrence with a follow up between 6 and 69 months. In the second series [14], 5 patients were asymptomatic, and 2 had got recurrence, with a follow up between 3 and 102 months.

**Table 1:** Review of the literature

**Year**	**Authors**	**Number of cases**
1982	Tasça and col. Benign peritoneal mesothelioma. Hystopathology in a case. Morphol Embryol; 28 (1): 47-9	1
1982	Katsube Y and col. Cystic mesothelioma of the peritoneum: a report of 5 cases and review of the literature. Cancer Oct 15; 50 (8)	5
1983	Schneider V and col. Benign cystic mesothelioma involving the female genital tract: report of four cases. Am J Obstet Gynecol; Feb 1; 145 (3)	4
1984	Philip G and col. Benign cystic mesothelioma. Case reports. British journal of Obstetrics and Gynaecology, Vol. 91, pp 932-938	2
1987	Pastormalo M and col. Benign cystic mesothelioma of the peritoneum. Minerva Ginecologia, Mar 39 (3)	1
1989	Betta PG and col. Benign cystic mesothelioma of the peritoneum. G Ital Oncol. Jan Mar; 9 (1)	1
1990	Hidvegi J and col. Benign cystic mesothelioma of the peritoneum. Orv Hetil. Feb 4; 131 (5)	1
1990	Chen YC and col. Benign cystic mesothelioma of the peritoneum: report of a case. J Formos Med Assoc. Jun; 89 (6)	1
1991	Hidvegi J and col. Peritoneal benign cystic mesothelioma. Pathol Res Pract. Jan; 187 (1)	1
1991	Pollack CV and col. Benign cystic mesothelioma presenting as acute abdominal pain in a young woman. J Emerg Med: 9 Suppl 1:21-5	1
1994	Kyzer S and col. Benign cystic mesothelioma of the peritoneum. Eur J Surg. May; 160 (5)	1
1995	Ricci F and col. Benign cystic mesothelioma in a male patient: surgical treatment by the laparoscopic route. Surg Laparosc endosc. Apr; 5 (2)	1
1995	Takenouchi Y and col. Report of a case of benign cystic mesothelioma. Am J Gastroenterol; Jul 90 (7)	1
1996	Tomasini P and col. Benign peritoneal multicystic mesothelioma. J Radiol; Jan 77 (1)	1
1996	Yaegachi N and col. Multilocular peritoneal inclusion cysts. J Obstet Gynaecol Res; Apr 22 (2)	1
1997	Cusatelli P and col. Benign cystic mesothelioma of the peritoneum: a case report. Eur J Gynaecol Oncol. 18 (2)	1
1997	Van Der Klooster and Col. Successful catheter drainage of recurrent benign multicystic mesothelioma of the peritoneum. Neth J Med, Jun; 50 (6)	1
1998	Abino JF and col. Peritoneal benign polycystic mesothelioma. Press Med, Apr 25; 27 (16)	1
1998	Letterie GS and col. The antiestrogen tamoxifen in the treatment of recurrent benign cystic mesothelioma. Gynecol Oncol, Jul; 70 (1)	1
1998	Kumar D and col. Benign cystic peritoneal mesothelioma in a man. Indian J Gastroenterol, Oct-Dec; 17 (4)	1
1999	Keiri-Vassilatou E and col. Benign cystic mesothelioma of the peritoneum an immunopathological study of three cases. Eur J Gyneacol Oncol. 20 (4)	3
1999	Jovovic M and col. Multicystic mesothelioma of the peritoneum. Vojnosanit Pregl. Mar-Apr; 56 (2)	1
1999	Park BJ and col. Treatment of primary peritoneal mesothelioma by continuous hyperthermic peritoneal perfusion (CHPP). Ann Surg Oncol, Sep;6(6):582-90.	18
2001	Petrou G and Col. Benign cystic mesothelioma in a 60 year old woman after cholecystectomy. ANZ J Surg, Oct; 71 (10)	1
2002	Hafner M and Col. Giant Benign cystic mesothelioma: a case report and review of the littérature. Eur J Gastroenterol Hepatol. 2002 Jan;14(1):77-80.	1
2002	Van ruth S and Col. Peritoneal Benign cystic mesothelioma: a case report and review of the literature. Eur J Surg Oncol. 2002 Mar;28(2):192-5	1
2002	Adolph AJ and col. Benign multicystic mesothelioma: a case report. J Obstet Gynaecol Can. 2002 Mar;24(3):246-7.	1
2002	Cavallaro A and col. Benign multicystic mesothelioma of the peritoneum: a case report. Chir Ital. 2002 Jul-Aug;54(4):569-72	1
2003	Shawn RN and col. Benign cystic mesothelioma of the peritoneum: a clinicopathologic study of 17 cases and immunohistochemical analysis of estrogen and progesterone receptor status. Hum Pathol. 2003 Apr;34(4):369-74.	17
2003	Bruni R and col. Benign cystic mesothelioma with multiple recurrences: a clinical case. Chir Ital. 2003 Sep-Oct;55(5):757-60	1
2004	Varma R and Col. Multicystic benign mesothelioma of the peritoneum presenting as postmenopausal bleeding and a solitary pelvic cyst--a case report. Gynecol Oncol. 2004 Jan;92(1):334-6.	1
2004	Baeyens P and col. Benign cystic peritoneal mesothelioma. JBR-BTR. 2004 May-Jun;87(3):114-5	1
2005	Szöllósi A and col. Benign cystic mesothelioma, a rare tumor of the peritoneum. Magy Seb. 2005 Feb;58(1):35-7	1
2005	Urbańczyk K and col. Mesothelial inclusion cysts (so-called benign cystic mesothelioma)--a clinicopathological analysis of six cases. Pol J Pathol. 2005;56(2):81-7.	6
2006	Svetlana M and col. Benign cystic mesothelioma of the peritoneum. Isr Med Assoc J. 2006 Jul;8(7):511-2	1
2006	Safioleas MC and col. Benign multicystic peritoneal mesothelioma: a case report and review of the literature.World J Gastroenterol. 2006 Sep 21;12(35):5739-42	New case: 1 Review: 130 cases
2007	Coskun A and col. Benign cystic mesothelioma presenting as a huge pelvic mass--a case report. Eur J Gynaecol Oncol. 2006;27(6):621-2	1
2007	Saad S and col. Benign peritoneal multicystic mesothelioma diagnosed and treated by laparoscopic surgery. J Laparoendosc Adv Surg Tech A. 2007 Oct;17(5):649-52	1
2008	Ashqar S and col. Benign mesothelioma of peritoneum presenting as a pelvic mass.J Coll Physicians Surg Pak. 2008 Nov;18(11):723-5	1
2008	Chammakhi-Jemli C and col. Benign cystic mesothelioma of the peritoneum. Tunis Med. 2008 Jun;86(6):626-8	1
2008	Stroescu and col. Recurrent benign cystic peritoneal mesothelioma. Chirurgia (Bucur). 2008 Nov-Dec;103(6):715-8	1
2009	Uzum N and col. Benign multicystic peritoneal mesothelioma.Turk J Gastroenterol. 2009 Jun;20(2):138-41	1
2010	Limone A and col. Laparoscopic excision of a benign peritoneal cystic mesothelioma. Arch Gynecol Obstet. 2010 Mar;281(3):577-8	1
2010	Pitta X and col. Benign multicystic peritoneal mesothelioma: a case report. J Med Case Rep. 2010 Nov 29;4:385	1
2011	Akbayir O and col. Benign cystic mesothelioma: a case series with one case complicated by pregnancy. J Obstet Gynaecol Res. 2011 Aug;37(8):1126-31.	3
2012	Lari F and col. Benign multicystic peritoneal mesothelioma. A case report. Recenti Prog Med. 2012 Feb;103(2):66-8	1
2012	Stojsic Z and col. Benign cystic mesothelioma of the peritoneum in a male child.J Pediatr Surg. 2012 Oct;47(10):e45-9	1
2012	Khuri S and col. Benign cystic mesothelioma of the peritoneum: a rare case and review of the literature. Case Rep Oncol. 2012 Sep;5(3):667-70.	1
2013	Singh A and col. Multicystic peritoneal mesothelioma: not always a benign disease.Singapore Med J. 2013 Apr;54(4):e76-8	1

## Conclusion

Benign cystic mesothelioma of the peritoneum (BCM) is a rare tumor with a high local recurrence rate. It requires optimal care in a specialized center especially as there is no evidence-based treatment strategies.

### Consent

Written informed consent was obtained from the patient for publication of this Case report and any accompanying images.

## Competing interests

All authors declare that they have no competing interests.

## Authors’ contributions

EBH and AB participated in writing the case report and revising the draft, OM, EB, AO, KM and KAT participated in the follow up. All authors read and approved the final manuscript.
